# Research on sound quality of roller chain transmission system based on multi-source transfer learning

**DOI:** 10.1038/s41598-024-62090-3

**Published:** 2024-05-16

**Authors:** Jiabao Li, Lichi An, Yabing Cheng, Haoxiang Wang

**Affiliations:** School of Mechanical and Aerospace Engineering, Jilin University, Changchun, China

**Keywords:** Transfer learning, Muti-source domain, Sound quality, Roller chain, Engineering, Mechanical engineering

## Abstract

To establish the sound quality evaluation model of roller chain transmission system, we collect the running noise under different working conditions. After the noise samples are preprocessed, a group of experienced testers are organized to evaluate them subjectively. Mel frequency cepstral coefficient (MFCC) of each noise sample is calculated, and the MFCC feature map is used as an objective evaluation. Combining with the subjective and objective evaluation results of the roller chain system noise, we can get the original dataset of its sound quality research. However, the number of high-quality noise samples is relatively small. Based on the sound quality research of various chain transmission systems, a novel method called multi-source transfer learning convolutional neural network (MSTL-CNN) is proposed. By transferring knowledge from multiple source tasks to target task, the difficulty of small sample sound quality prediction is solved. Compared with the problem that single source task transfer learning has too much error on some samples, MSTL-CNN can give full play to the advantages of all transfer learning models. The results also show that the MSTL-CNN proposed in this paper is significantly better than the traditional sound quality evaluation methods.

## Introduction

Studies have shown that long-term exposure to noise can seriously affect people’s mental and physical health. Mechanical noise not only makes people cranky, but also can damage hearing and even lead to a higher risk of heart disease^1,2^. As an important mechanical basic product, roller chain is widely used in automobiles and motorcycles, which directly affects the noise quality of the whole machine. Nowadays, users pursue higher noise comfort, so how to evaluate the noise of the roller chain transmission system is particularly important. However, previous studies on roller chain noise mainly focus on noise characteristics and noise generation mechanism^3,4^. Due to the lack of relevant research on sound quality, a series of noise tests are carried out in this paper to establish the sound quality evaluation model of the roller chain system.

Sound quality research generally includes subjective and objective evaluation content, exploring the user's subjective feeling to noise, mainly the evaluation of comfort degree. The research on sound quality mainly focuses on the field of automobile, and researchers generally use some acoustic parameters (A-weighted sound pressure level, loudness, sharpness, roughness, fluctuation, articulation index and tonality) as objective evaluation. Based on the specific subjective evaluation method, the testers are organized to conduct the auditory evaluation test on the noise samples. Then, researchers often use some machine learning and neural network methods to establish a sound quality evaluation model with objective evaluation as input and subjective evaluation as output^5–7^. Li, D. et al. used the method of multiple linear regression to establish the relationship between subjective discomfort degree and acoustic parameters. The results show that loudness and sharpness have the greatest influence on the comfort of micro commercial vehicles^8^. Wang, Y. et al. proposed a global annoyance level modeling method for pure electric vehicles and established a nonlinear mapping relationship between psychoacoustic indicators and sound quality based on the extreme gradient boost algorithm^9^. Sometimes there is severe multicollinearity between the input features, and the accuracy and performance of the sound quality evaluation model will be reduced. Convolutional neural network (CNN) has strong feature extraction ability and is generally used in the field of image processing. In order to use CNN in the study of sound quality, various feature maps are constructed as objective evaluation of noise^10,11^. Mel frequency cepstral coefficient (MFCC) is a commonly used feature representation method in sound signal processing, which has been proved to be used to distinguish sound quality^12^. Using the MFCC feature map as the input of the model, CNN is obviously better than the traditional sound quality modeling method. The above research introduces the development of sound quality research and the improvement of modeling methods in different application scenarios. However, how to improve the accuracy of sound quality prediction model is still a difficult problem when the number of samples is insufficient. Moreover, the constant pursuit of prediction accuracy will inevitably lead to more and more complex models, so it is also important to achieve lightweight sound quality prediction models.

To establish the sound quality evaluation model of roller chain transmission system, we first collect the running noise of the roller chain system under different working conditions and preprocess the noise samples. Secondly, we organize a group of testers to evaluate the noise samples subjectively, and construct feature maps as objective evaluation. To solve the problem of small sample sound quality evaluation, we find and make use of a fuzzy phenomenon in the subjective evaluation of sound quality and propose a data enhancement method called fuzzy generation. However, fuzzy generation can lead to a particular kind of data leakage, and we use transfer learning to eliminate this effect. The basic idea of transfer learning is to accelerate the learning process on the target task by using the knowledge learned in one or more source tasks. This is usually done by using a model that has already been trained on one task as a starting point, and then fine-tuning it to fit the new task^13–16^. In the study of single-source tasks, Jamil, F. et al., proposed an example-based deep transfer learning method for wind turbine gearbox fault detection to prevent negative migration^17^. Maschler, B. et al. proposed a modular deep learning algorithm for anomaly detection of time series data sets, which realizes deep industrial transfer learning^18^. In the study of multi-source tasks, Rajput, D. S. et al. use multi-source sensing data and fuzzy convolutional neural network for fault classification and prediction, and the accuracy of the model is significantly superior to other machine learning and deep learning methods^19^. Sun, L. et al., proposed a new parameter transfer method to improve training performance in multi-task reinforcement learning^20^. Based on the sound quality study of three chain transmissions (silent chain, Hy-Vo chain and dual-phase Hy-Vo chain), we propose a new method named multi-source transfer learning convolutional neural network (MSTL-CNN). The results show that the proposed model is superior to the traditional sound quality prediction methods.

## Data collection and pre-processing

**Figure 1:**
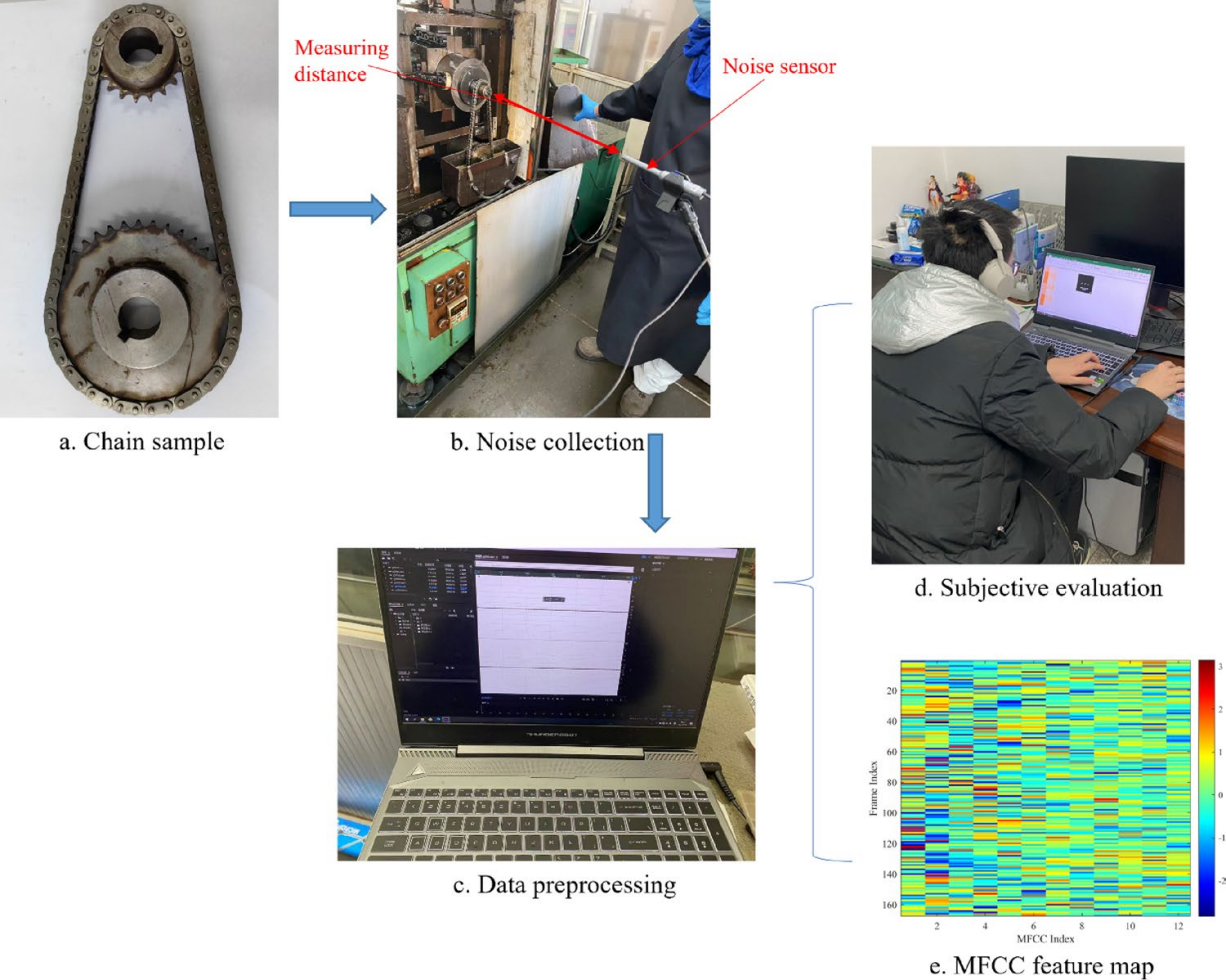
Test procedure.

Figure 1 shows the steps of the sound quality evaluation test, including noise acquisition and processing, subjective evaluation test and objective evaluation.

### Noise test

For the chain transmission system, the roller chain type is 06B, the tooth number of driving sprocket is 19, and the tooth number of driven sprocket is 38. The noise sensor (MINIDSP UMIK-1) is placed at the same height as the center of the driving sprocket, and the noise test is carried out in a closed indoor reverberation field. The measurement distance is the distance between the noise sensor and the chain system, which is 0.5 and 1 m respectively. In the noise test, the speed range is 500–4000 rpm, and the three loads are 500 N, 600 N and 750 N respectively. Starting from the lowest speed (500 rpm), the noise collection is performed every 500 rpm, and the collection time is greater than 30 s each time. We record noise audio using Adobe Audition 2022 software, and the noise is sampled at 48,000 Hz. Finally, we can get 2 × 8 × 3 = 48 noise samples, and randomly intercept 5 s fragments of each sample for subsequent processing.

**Figure 2:**
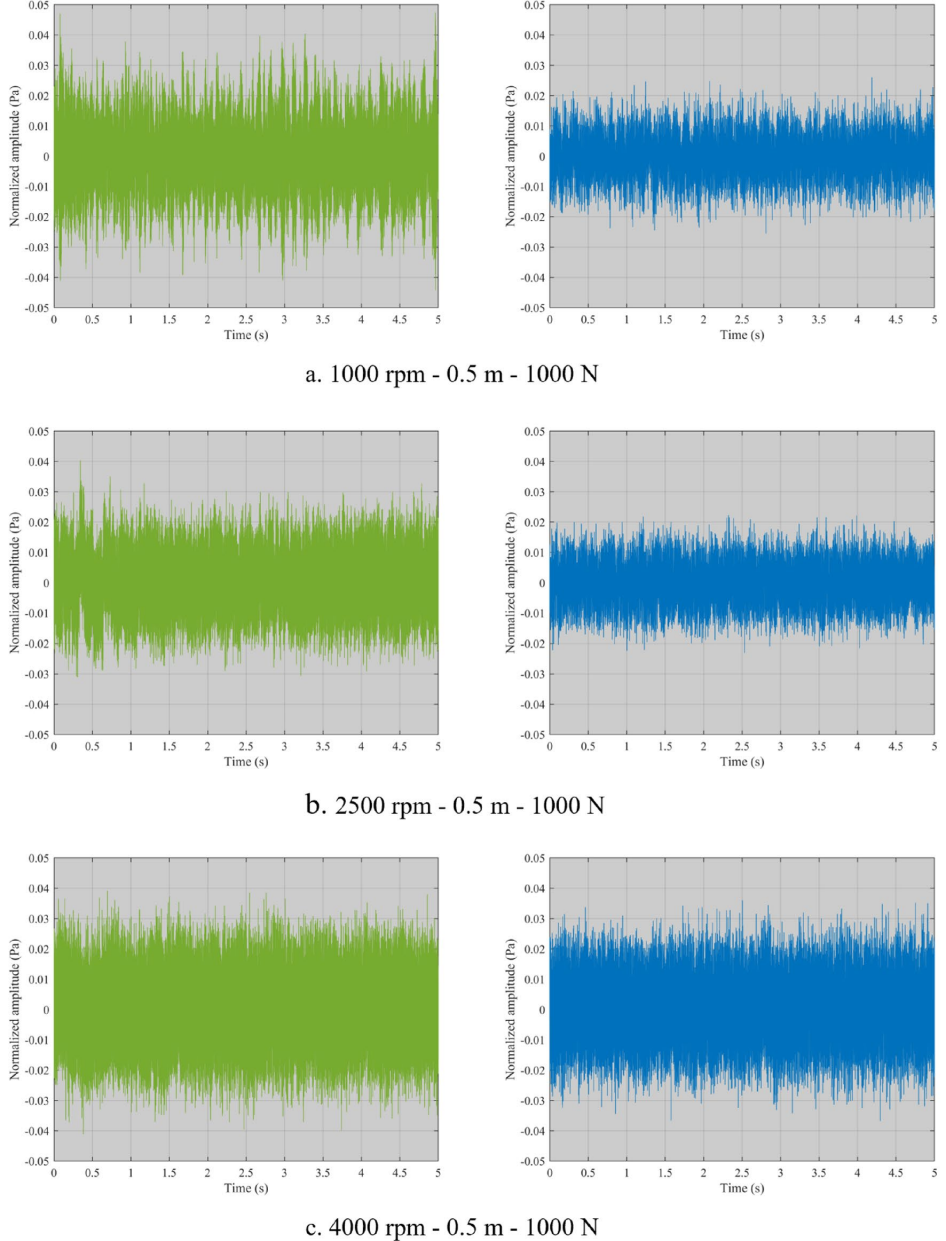
Comparison of time domain waveform.

Figure 2 shows a comparison between the noise of the roller chain transmission system and a silent chain transmission system. The green time-domain waveform on the left is the roller chain system, and the blue one on the right is the silent chain system. Under the same load and measurement distance, the noise of the roller chain system is stronger than that of the silent chain from low speed to high speed. At the low-speed of 1000 rpm, the noise energy of the roller chain system is obviously stronger than that of the silent chain. As the speed increases, the noise energy of the roller chain system decreases at 2500 rpm. The results show that the roller chain system works more smoothly at medium speed, but the noise is still stronger than that of the silent chain system. At the high speed of 4000 rpm, the noise energy of both increases significantly, and the noise of the roller chain system is slightly stronger than that of the silent chain system. Therefore, the noise characteristics of the roller chain system are different from those of the silent chain system, so it is necessary to construct the sound quality evaluation model of the roller chain transmission system.

### Subjective evaluation test

**Figure 3:**
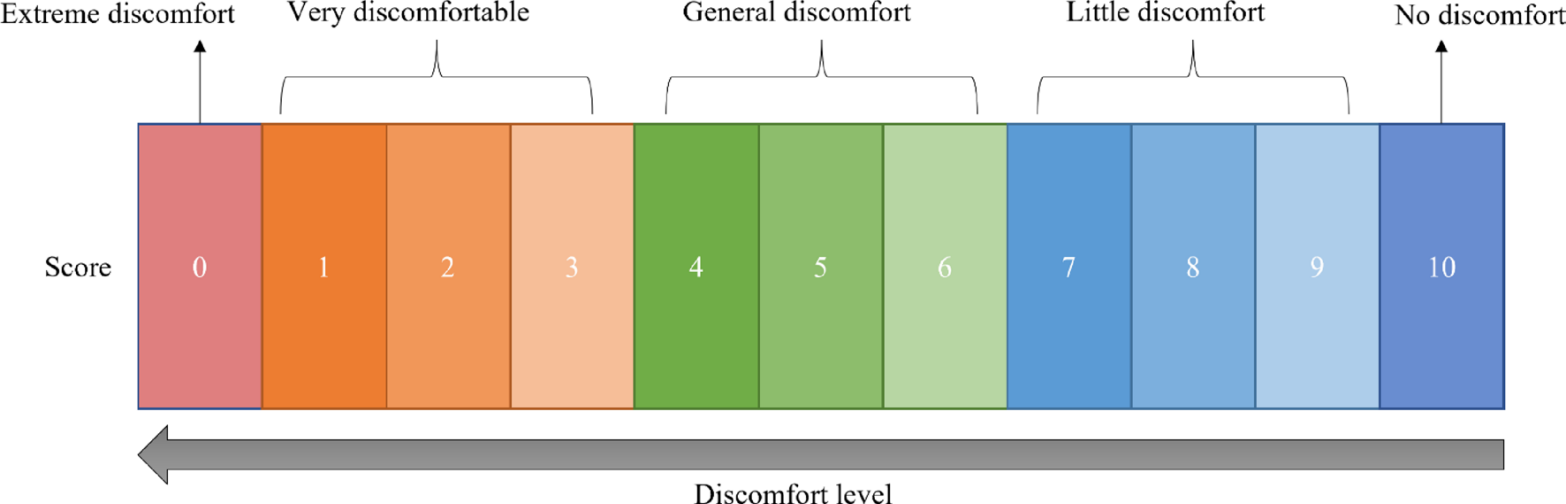
Subjective evaluation score chart.

We use the equal interval direct one-dimensional evaluation as a subjective evaluation method, and the noise discomfort level describes the sound quality^21^. As shown in Fig. 3, there are five discomfort levels for noise, where 0 means extreme discomfort and 10 means no discomfort. The three middle discomfort levels, with three scores for each level, represent the strength of the discomfort level from smallest to largest. Twelve healthy testers with driving experience take part in the auditory perception test, with a ratio (5:1) of men to women. As shown in Fig. 1d, the tester wears a hi-fi headset for the test, and the same noise audio is played five times by the Groove software. The sound pressure level of the test environment does not exceed 30 dB, and the score is recorded in the table after the tester listens to a noise audio.

**Figure 4:**
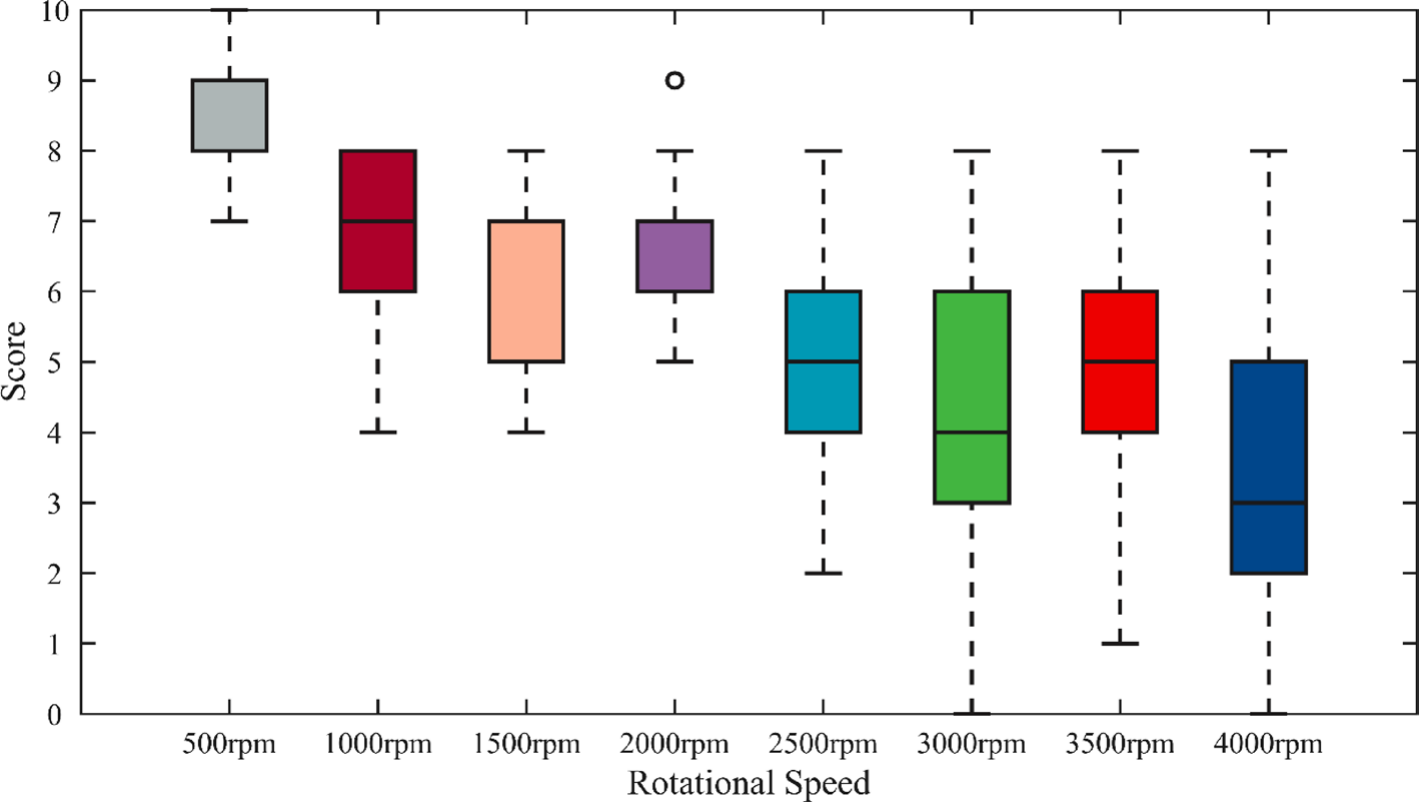
Box plot of scores.

The subjective evaluation scores at each speed are shown in Fig. 4. Based on the median line, as the speed continues to increase, the score overall shows a downward trend. In the speed range of 500–3000 rpm, the sound quality of the roller chain system decreases with the increase of the speed. However, at 3500 rpm, the score increases, indicating that the roller chain system runs more smoothly at this time. At the limit speed of 4000 rpm, the score is significantly reduced. It should also be noted that the longer the box line means that the sound quality of this speed fluctuates greatly, and there is even an outlier at 2000 rpm. For the same sample, twelve testers should have relatively consistent feelings, so it is necessary to conduct normality test and correlation test on the subjective evaluation results.

**Figure 5:**
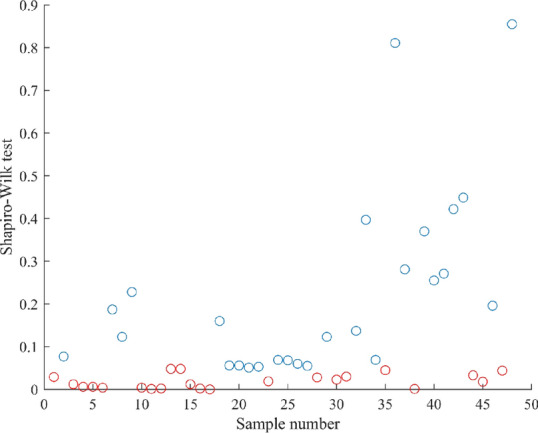
Normality test.

**Figure 6:**
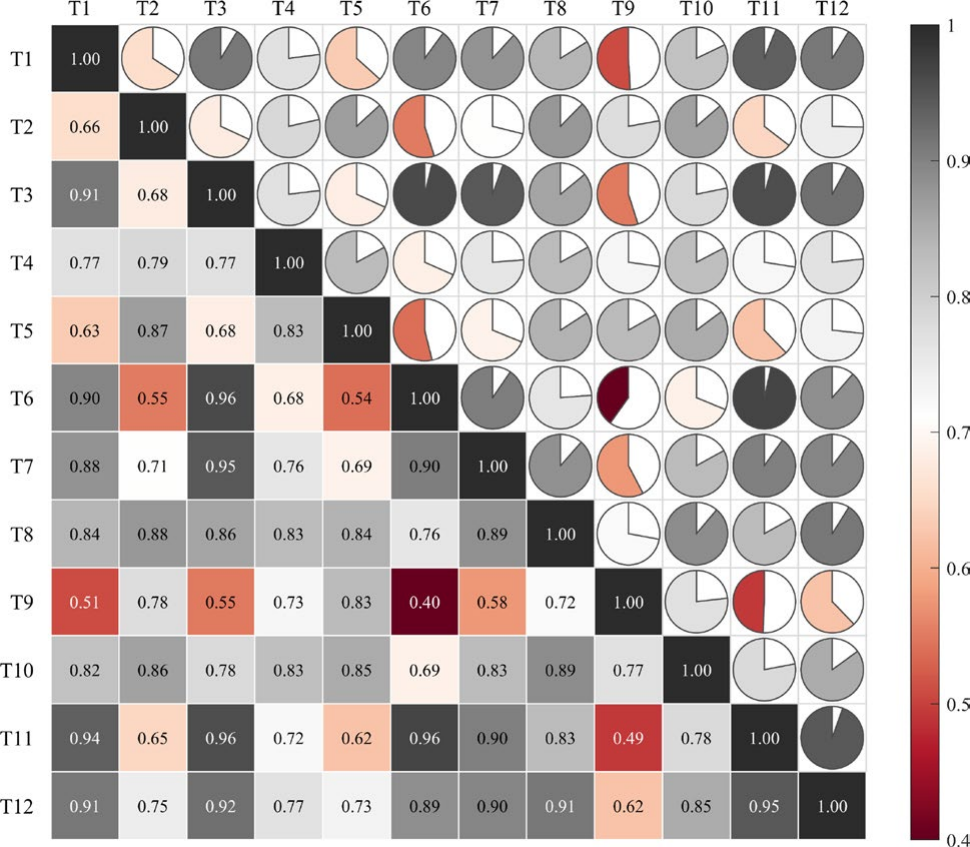
Correlation test result.

Because the sample size is small, Shapiro–Wilk test is used to test the normality of each sample scores, as shown in Fig. 5. If the test value is greater than 0.05, it is consistent with normality, which is represented by the blue circle, and the red circle indicates that the sample does not conform to the normal distribution. We can see that quite a variety of evaluation results do not conform to the normal distribution, so spearman correlation test is needed to further analyze the subjective evaluation results. The formula for spearman correlation test is as follows:

1 $$r = \frac{{\sum\limits_{i = 1}^{n} {(x_{i} - \overline{x})\left( {y_{i} - \overline{y}} \right)} }}{{\sqrt {\sum\limits_{i = 1}^{n} {(x_{i} - \overline{x})^{2} \sum\limits_{i = 1}^{n} {(y_{i} - \overline{y})^{2} } } } }}$$

where *x*_*i*_ and *y*_*i*_ represent the corresponding elements of the two variables, $\overline{x}$ and $\overline{y}$ represent the average value of the corresponding variables. The larger the *r* value, the stronger the correlation, and the test results are shown in the Fig. 6.

In Fig. 6, T_*i*_ (*i* = 1, 2,…,12) represents the number of the twelve testers. The correlation between many testers is less than 0.7, and the weakest correlation between T9 and T6 is only 0.40. To screen reasonable subjective evaluation results, we calculated the average correlation coefficient (ACC) of each tester based on Fig. 6, as shown in Table 1.

**Table 1:** ACC of twelve testers.

Tester	T1	T2	T3	T4	T5	T6	T7	T8	T9	T10	T11	T12
ACC	0.80	0.74	0.82	0.77	0.74	0.75	0.82	0.84	0.63	0.81	0.80	0.84

It can be seen from Table 1 that the ACC of T9 is less than 0.7, so if the results of T9 are excluded, the evaluation results of the remaining testers are all reasonable. The average evaluation scores of the remaining eleven testers are calculated as the final subjective evaluation results, as shown in Table 2.

**Table 2:** Final score of each sample.

Sample number	1	2	3	4	5	6	7	…	42	43	44	45	46	47	48
Score	8.82	8.73	9.00	8.82	8.82	8.91	6.55	…	4.36	2.73	2.09	1.00	5.55	4.55	2.73

### Objective evaluation

Mel frequency cepstral coefficient (MFCC) is based on the characteristics of the human auditory system. The sensitivity of the human ear to different frequencies is non-linear: it is more sensitive to low frequency sounds and less sensitive to high frequency sounds. The Mel scale is a frequency measurement method based on the human ear’s perception of pitch, which can convert the actual frequency into the Mel frequency. MFCC is a powerful feature representation method because it is able to capture the main characteristics of speech signals in a compact manner, and to some extent simulates the characteristics of human auditory perception^22–24^. The calculation steps of MFCC are as follows:

1. The original signal is pre-weighted, the high-frequency part is strengthened, and the high-frequency part of the sound signal is compensated for the loss that may be suffered during transmission.
  2 $$y(t) = x(t) - \alpha x(t - 1)$$
  where *x*(*t*) is the original signal, *y*(*t*) is the pre-weighted signal, and *α* usually takes 0.95 or 0.97.
2. The signal is divided into *N* millisecond frames, and the data of each frame is windowed. Window functions usually use Hamming window:
  3 $$\omega (n) = 0.54 - 0.46 \cdot \cos (2\pi n/N) \, 1 < n < N$$
3. The frequency spectrum is obtained by fast Fourier transform of the data of each frame. A set of Mel filters (usually a triangular filter bank) is applied to the spectrum to simulate the perceptual properties of the human ear. Each filter *H*_*m*_(*k*) is defined as:
  4 $$H_{m} (k) = \left\{ {\begin{array}{*{20}l} 0 \hfill & {{\text{k < f}}(m - 1)} \hfill \\ {\frac{k - f(m - 1)}{{f(m) - f(m - 1)}}} \hfill & {{\text{f}}(m - 1) \le k \le f(m)} \hfill \\ {\frac{f(m + 1) - k}{{f(m + 1) - f(m)}}} \hfill & {{\text{f}}(m) \le k \le f(m + 1)} \hfill \\ 0 \hfill & {{\text{k}} \ge {\text{f}}(m + 1)} \hfill \\ \end{array} } \right.$$
  where *f*(*m*) is the central frequency of the filter on the Mel scale. The Mel scale transformation is shown as follows:
  5 $$M(f) = 2595 \cdot \log_{10} \left( {1 + \frac{f}{700}} \right)$$
  where *M*(*f*) is the representation of the frequency *f* on the Mel scale.
4. The output of the filter bank needs to be logarithmic.
  6 $$S(m) = \log \left( {\sum\nolimits_{k = 0}^{K - 1} {\left| {X(k)} \right|^{2} \cdot H_{m} (k)} } \right)$$
  where *X*(*k*) is the spectrum of the frame.
5. After taking the logarithm of the filter bank output, the discrete cosine transform is used to get the final MFCC.
  7 $$C(n) = \sum\nolimits_{m = 0}^{M - 1} {S(m) \cdot \cos \left[ {\frac{\pi n}{M}(m + \frac{1}{2})} \right]} {,}\quad n = 1,2,...,L$$
  where *C*(*n*) is the *n*-th MFCC,* L* represents the order of the MFCC, generally 12–16.

**Figure 7:**
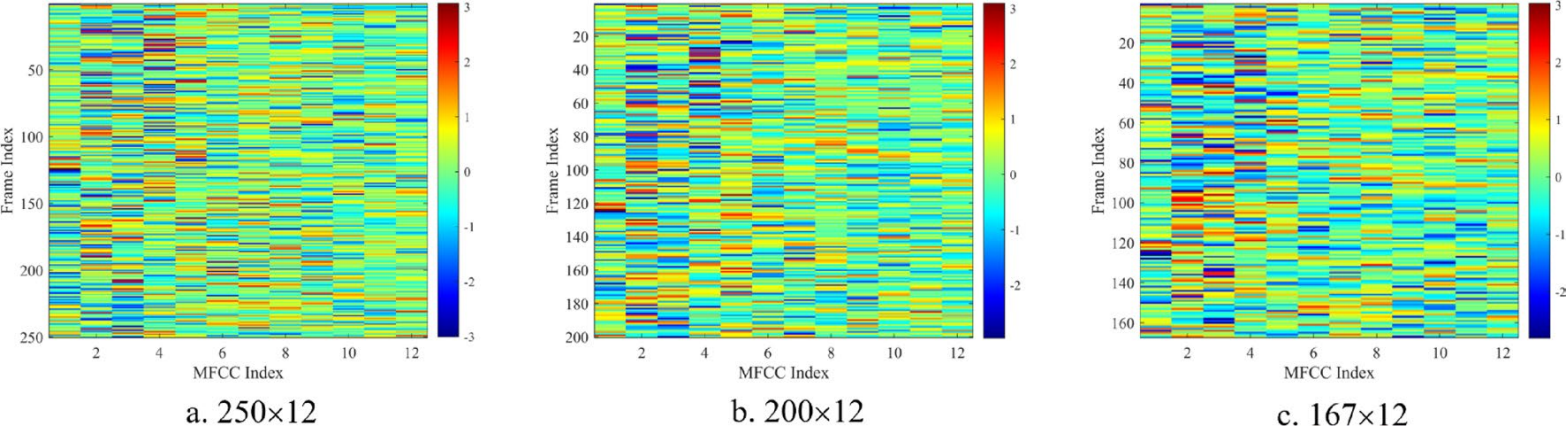
MFCC feature map.

**Figure 8:**
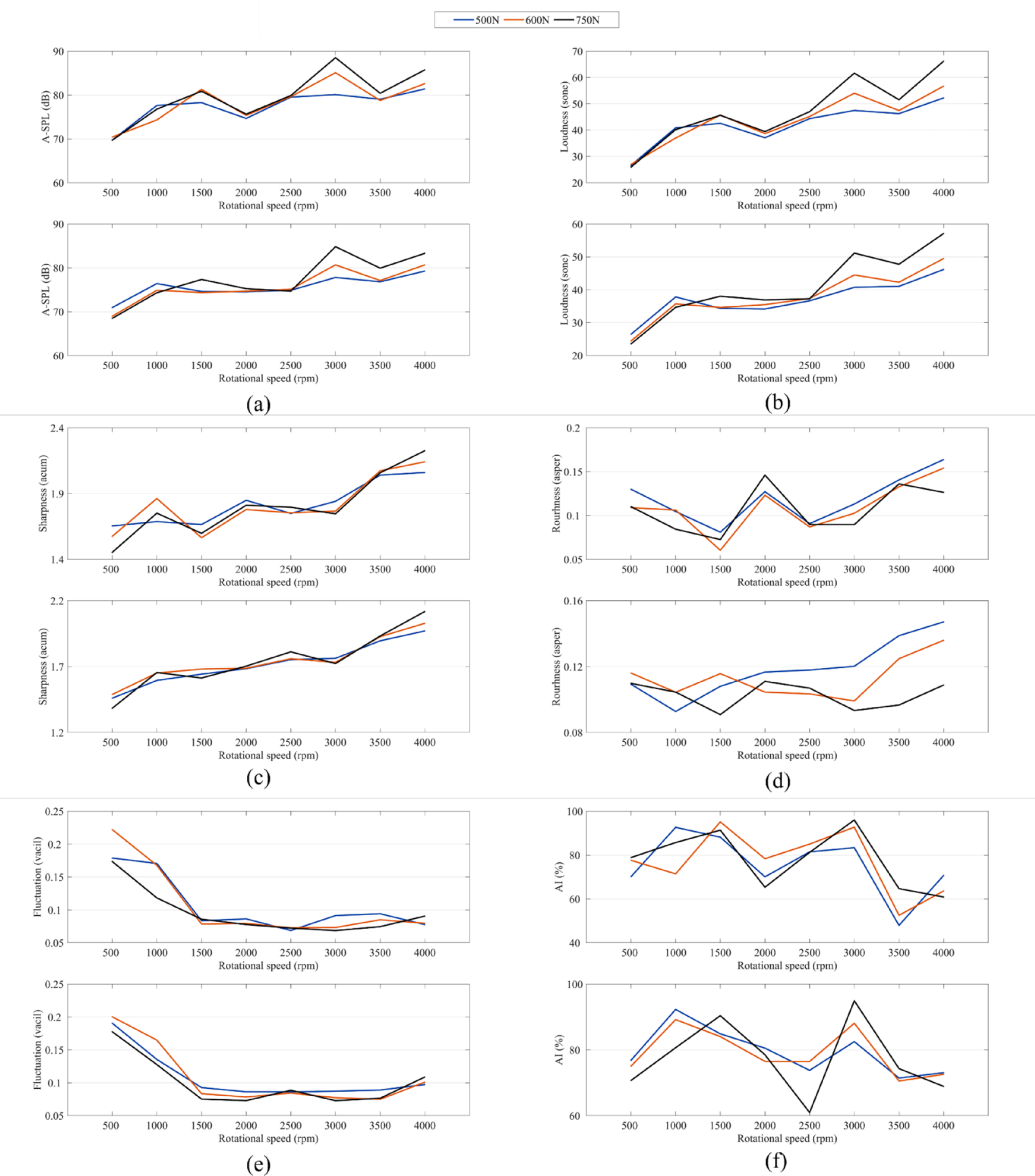
Six acoustic parameters: (**a**) A-SPL, (**b**) loudness, (**c**) sharpness, (**d**) roughness, (**e**) Fluctuation, (**f**) AI (The upper figure of each subgraph represents the parameters at 0.5 m measuring distance and the lower figure of each subgraph represents the parameters at 1 m measuring distance).

In this paper, the MFCC order *L* is taken as 12, and the length *N* of each frame is taken as 20 ms, 25 ms and 30 ms respectively. Finally, the 5 s noise sample is divided into *F* (250,200 and 167) frames. Based on Eqs. (2)–(7), we can calculate the three MFCC for each noise sample. The objective evaluation results generate the input feature space, and the subjective evaluation labels the noise samples. As shown in Fig. 7, the MFCC feature map is constructed as the input of the sound quality evaluation model. To compare with the traditional modeling method of sound quality evaluation, we also select six acoustic parameters as objective evaluation. As illustrated in Fig. 8, the Audio toolbox in MATLAB is used to calculate these six parameters: A-weighted sound pressure level (A-SPL), loudness, sharpness, roughness, fluctuation, articulation index (AI).

## Methodology

Transfer learning (TL) is especially useful in situations where data is scarce, because it allows models to leverage existing knowledge, reducing the need for large amounts of labeled data. TL can be divided into four categories according to different technical methods: instance-based TL, feature-based TL, model-based TL and relation-based TL^25,26^. Instance-based TL directly uses the data instances of the source task to assist the learning of the target task, which usually involves reweighting the data of the source task to better adapt to the target task. Feature-based TL learns feature representations that can be transferred between source and target tasks. Model-based TL directly transfers the model parameters of the source task to the target task and adjusts them. Relation-based TL is suitable for situations where both source and target tasks involve relational data, such as knowledge graphs or social networks. These classification methods of TL help to understand its wide application and provide guidance for selecting appropriate transfer learning strategies for specific problems.

**Figure 9:**
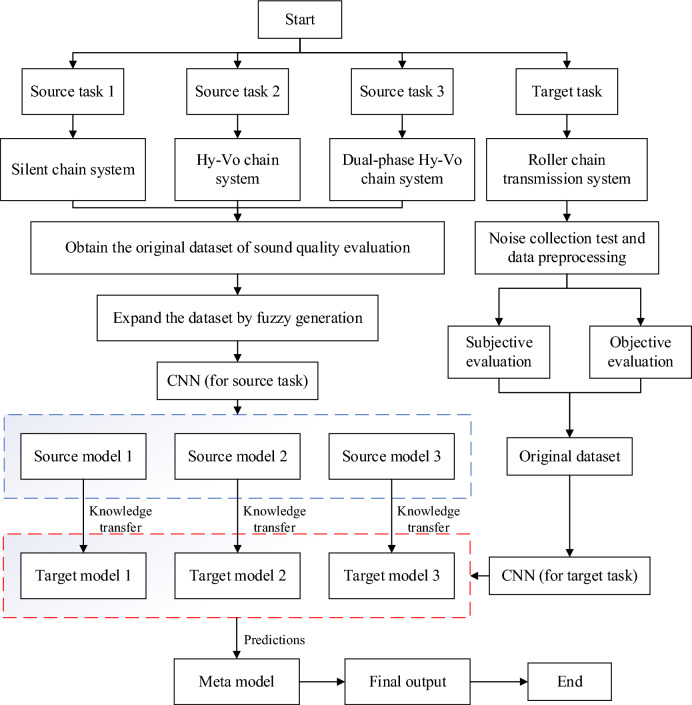
MSTL-CNN flowchart.

In this paper, we take the sound quality evaluation of roller chain transmission system as the target task and choose the sound quality study of silent chain transmission system as the source task. The source task and the target task are the same in feature space and data space, but the data distribution is different. We choose the model-based TL approach, in which the model parameters of the source task are used as the initialization parameters of the target task model^27^. The fine-tuning process can be represented by the following formula:

8 $$\theta_{{{\text{target}}}} = \theta_{{{\text{source}}}} + \Delta \theta$$

where *θ*_target_ is the model parameter of the target task, *θ*_source_ is the model parameter of the source task, and Δ*θ* is the parameter adjustment on the target task. As shown in Fig. 9, by stacking the prediction results of the three target models, the final sound quality evaluation model can be obtained.

**Figure 10:**
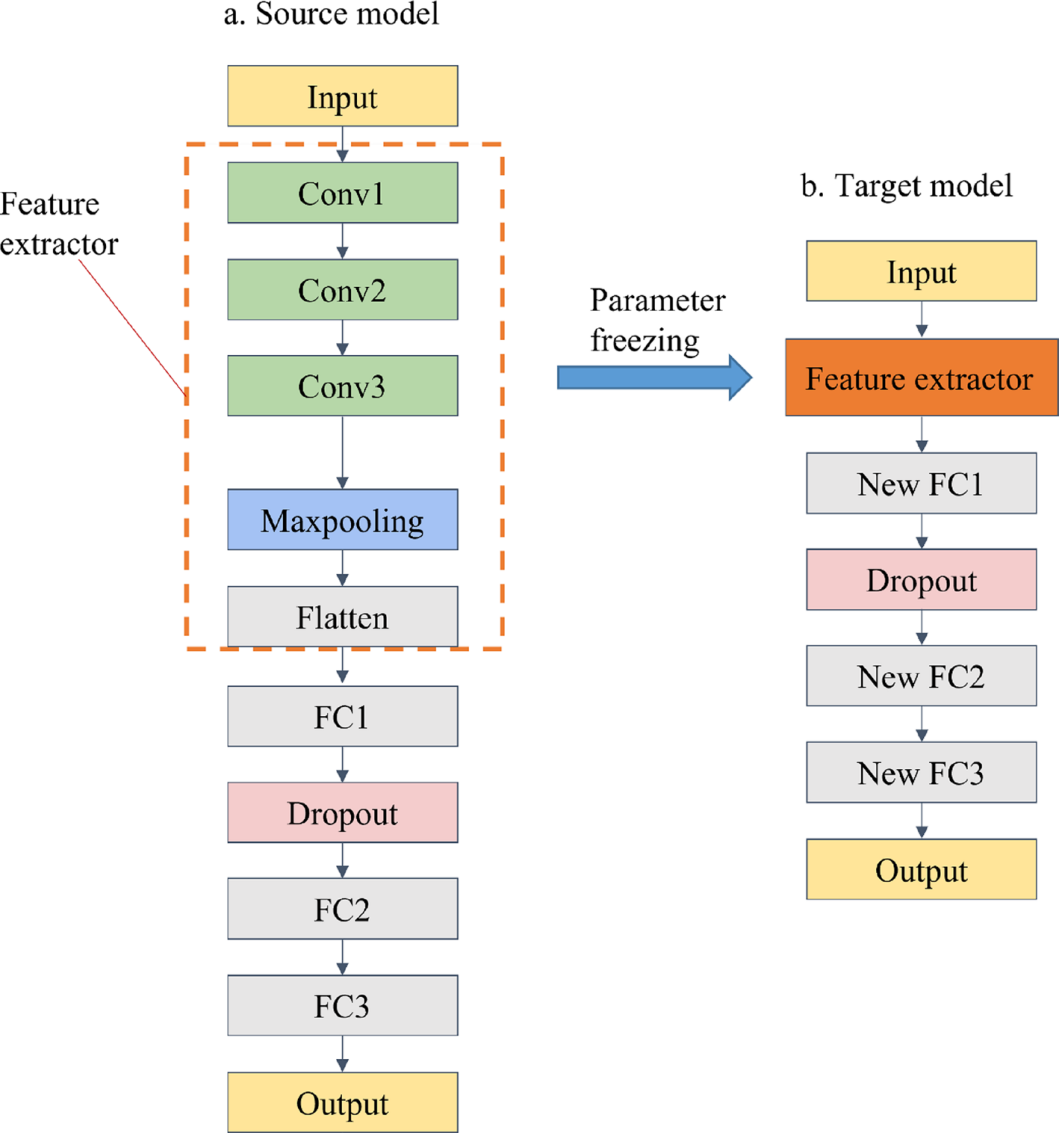
Transfer learning model.

As a basic model, convolutional neural network (CNN) is generally used to solve classification tasks. To model sound quality evaluation, the number of nodes in the output layer is set to 1, and a continuous value can be obtained without using nonlinear activation function. The convolutional layer in front of the CNN can be regarded as a feature extractor, as shown in the Fig. 10. In the source model, there are three convolution layers (Conv), one maxpooling layer, one flatten layer, and three fully connected layers (FC). The step size of the maximum pooling layer is 2 with 0 padding, and the step size of the Conv is 1 without 0 padding. In the three FC, the number of nodes is 1024, 128, and 1, respectively. To avoid overfitting, dropout technology is used in FC1, and the dropout rate is set to 0.5. The output layer is the last layer, and the output result is the evaluation score. Except for the last layer, the activation functions of other layers are relu. Some studies have shown that the structure of the source model and the target model should be similar^28^. Therefore, in this paper, the structural parameters of the target model are the same as those of the source model. When the 167 × 12 MFCC feature map is used as input, the structural parameters of the source model are shown in the Table 3. The transfer learning process from the source model to the target model can be summarized as: First, the source model is trained on the source task, and then the feature extractor of the source model is reused on the target model. Based on the samples of the target task, the parameters of the new fully connected layers can be trained on the target model.

**Table 3:** Model structure parameters.

Layer type	Channels/units
Input 167 × 12	3
3 × 3 Conv relu, stride 1	4
3 × 3 Conv relu, stride 1	8
3 × 3 Conv relu, stride 1	12
2 × 2 Maxpooling relu, stride 2	48
Flatten	2916
FC1	1024
Dropout	1024
FC2	128
FC3	1

We chose three sound quality research of chain transmission system as the source tasks, which are: silent chain, Hy-Vo chain, and dual-phase Hy-Vo chain. To better train the source model, we need to expand the datasets for these three source tasks. Fuzzy generation is a data enhancement method based on the fuzzy phenomenon in the subjective evaluation of sound quality. After the correlation test, all the subjective evaluation scores are reasonable, but researchers often take the mean as the final score. If the average score is taken as the most correct result, the accuracy of the other scores can be defined. Based on the uncertainty of subjective evaluation results, we introduce fuzzy mathematics to quantify and deal with the fuzziness of this problem. Fuzzy mathematics is an effective mathematical tool for dealing with uncertainty and fuzziness. By introducing the concepts of fuzzy sets and fuzzy logic, it allows mathematical modeling of inaccurate or incomplete information that is prevalent in the real world^29–31^. Combined with the idea of fuzzy mathematics, fuzzy generation is proposed to expand the datasets. First, we can define a fuzzy map on the evaluation score interval as follows:

9 $$\begin{gathered} M:I \to \left[ {0,1} \right] \hfill \\ \, s \, \mapsto M(s) \hfill \\ \end{gathered}$$

where *I* is the value field [0 10], *M* is the fuzzy interval of *I*, and *M(s)* is the membership function.

**Figure 11:**
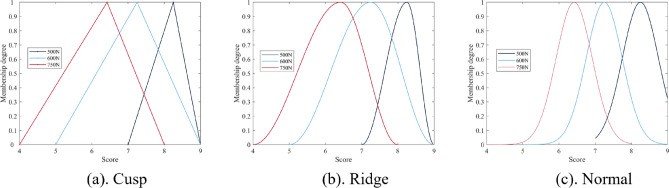
Three membership functions.

We treat the average score as having a membership of 1, while the minimum score and the maximum score both have a membership of 0. After constructing different membership functions, the membership degrees of different sizes are selected to divide the fuzzy generation interval. In the fuzzy generation interval, a suitable perturbation method is selected to generate a sufficient number of new samples. In this paper, three membership functions (cusp, ridge and normal) are constructed, and the formula is as follows:

10 $$M(s_{r} ) = \left\{ {\begin{array}{*{20}l} 0 \hfill & {{(0} \le r < m{)}} \hfill \\ {\frac{1}{c - m}(r - m)} \hfill & {{(}m \le r < c)} \hfill \\ {\frac{1}{n - c}(r - d)} \hfill & {{(}c \le r \le n)} \hfill \\ 0 \hfill & {{(}n < r \le 10{)}} \hfill \\ \end{array} } \right.$$

11 $$M(s_{r} ) = \left\{ {\begin{array}{*{20}l} 0 \hfill & {{(0} \le r < m{)}} \hfill \\ {\frac{1}{2}{ + }\frac{1}{2}\sin \frac{\pi }{c - m}\left[ {r - \frac{c + m}{2}} \right]} \hfill & {{(}m \le r < c)} \hfill \\ {\frac{1}{2} - \frac{1}{2}\sin \frac{\pi }{n - c}\left[ {r - \frac{c + n}{2}} \right]} \hfill & {{(}c \le r \le n)} \hfill \\ 0 \hfill & {{(}n < r \le 10{)}} \hfill \\ \end{array} } \right.$$

12 $$M(s_{r} ) = \left\{ {\begin{array}{*{20}l} 0 \hfill & {{(0} \le r < m{)}} \hfill \\ {e^{{ - 2(r - c)^{2} }} } \hfill & {{(}m \le r \le n)} \hfill \\ 0 \hfill & {{(}n < r \le 10{)}} \hfill \\ \end{array} } \right.$$

where *c* is the core point (the average score), *m* is the left boundary point (the minimum score), *n* is the right boundary point (the maximum score), *r* is a random generation point, and *M*(*s*_*r*_) is the membership of *r*. Equation (10) is the cusp membership function, Eq. (11) is the ridge membership function, and Eq. (12) is the normal membership function. We take three samples of the roller chain transmission system as an example, and the three membership functions are shown in Fig. 11. In this paper, we use random perturbation to triple the size of the original dataset with three membership degrees (0.9, 0.7 and 0.5).

## Results and analysis

Based on three source tasks (with 168 samples) and one target task, we build three transfer learning models: Source model 1(silent chain)–Target model 1, Source model 2(Hy-Vo chain)–Target model 2, Source model 3(dual-phase Hy-Vo chain)–Target model 3. On the source model and the target model, the MFCC feature map is input, the evaluation score is output. Since a large dataset is obtained through fuzzy generation, simple segmentation is enough to obtain reliable evaluation. Therefore, for the test method of source model and target model, we choose training-test split, that is, 83% of data sets are randomly selected for training and 17% for testing. Both the source model and the target model use the Adam optimizer, and the root mean squared error is the loss function. The initial learning rate of the source model is 0.005 and the epoch is set to 200. After each source model is trained on the source task, this part including the convolution layers, pooling layer, and flatten layer is regarded as the feature extractor and the parameters of these layers are frozen. By connecting the feature extractor with the new input layer, the new fully connected layers, the new dropout layer, and the new output layer, we can finally get three target models. For the target model, we use the dataset of the roller chain system for training, the initial learning rate is 0.003 and the epoch is set to 100. During the training process, only the parameters of the new fully connected layers are constantly updated, and with the help of the feature extractor, the model can converge with fewer epochs. The final results need to take the mean of five training results, and since there are 3 kinds of feature maps, 3 membership functions and 3 membership values, each transfer learning model needs to be trained 5 × 3 × 3 × 3 = 135 times in total. Three indicators: correlation coefficient (R), root mean squared error (RMSE) and mean absolute error (MAE) are selected to evaluate the transfer learning model, and the formulas are as follows:

13 $${\text{R}} = \frac{{\sum\nolimits_{i = 1}^{n} {(x_{i} - \overline{x})(y_{i} - \overline{y})} }}{{\sqrt {\sum\nolimits_{i = 1}^{n} {(x_{i} - \overline{x})^{2} } } \sqrt {\sum\nolimits_{i = 1}^{n} {(y_{i} - \overline{y})^{2} } } }}$$

14 $$RMSE = \sqrt {\frac{1}{n}\mathop \sum \nolimits_{i = 1}^{n} (x_{i} - y_{i} )^{2} }$$

15 $$MAE = \frac{1}{n}\mathop \sum \nolimits_{i = 1}^{n} \left| {x_{i} - y_{i} } \right|$$

where *n* is the number of samples, *x*_*i*_ is the predicted value of the sample, and *y*_*i*_ is the true value of the sample. *R* is used to measure the degree of linear correlation between two variables. The value is between − 1 and 1, where 1 means a completely positive correlation, − 1 means a completely negative correlation, and 0 means no linear correlation. In the prediction of sound quality, *R* can be used to intuitively show the linear correspondence between the predicted value of the model and the real value. To measure the final predictive effect of transfer learning, we also propose an evaluation formula to select the best situation.

16 $$E = \frac{{R_{{{\text{source}}}} }}{{R_{{{\text{target}}}} }}\left( {RMSE_{{{\text{target}}}} + MAE_{{{\text{target}}}} } \right)$$

The indicator *E* can represent the effectiveness of the transferred knowledge on the target model. To ensure that the performance difference between the source model and the target model is not too large, the value of *E* should be as small as possible. Based on the size of the* E* value, we can get the best set of source model-target model. Due to the small sample size of the target task, serious underfitting occurs in the model without the use of transfer learning, and the results are not presented because they are meaningless.

The training results of the Source model 1 are shown in Table 4, and the training results of the Target model 1 are shown in Table 5. The smaller the evaluation indicator *E*, the greater the contribution of the source model, and the smaller the prediction error of the target model. As Table 5 shows, the minimum value of evaluation indicator *E* is 1.745. Therefore, when the membership function is ridge, the membership value is 0.7 and the input size is 167 × 12, the transfer learning effect is the best. The training results of the Source model 2 and Target model 2 are presented in Tables 6 and 7 respectively.

**Table 4:** Results of source model 1.

Membership function	Membership value	Input size	Source model 1					
			Training			Test		
			R	RMSE	MAE	R	RMSE	MAE
Cusp	0.9	250 × 12	0.953	0.785	0.613	0.952	0.847	0.688
		200 × 12	0.963	0.749	0.609	0.969	0.767	0.640
		167 × 12	0.979	0.491	0.420	0.977	0.474	0.412
	0.7	250 × 12	0.954	0.718	0.568	0.953	0.747	0.627
		200 × 12	0.976	0.613	0.527	0.976	0.652	0.577
		167 × 12	0.971	0.485	0.372	0.974	0.475	0.398
	0.5	250 × 12	0.692	1.283	0.991	0.371	1.615	1.248
		200 × 12	0.714	1.217	0.920	0.392	1.648	1.253
		167 × 12	0.711	1.211	0.916	0.424	1.637	1.253
Ridge	0.9	250 × 12	0.951	0.802	0.646	0.939	0.793	0.670
		200 × 12	0.979	0.512	0.431	0.982	0.482	0.420
		167 × 12	0.968	0.594	0.472	0.971	0.598	0.497
	0.7	250 × 12	0.939	0.806	0.664	0.941	0.754	0.608
		200 × 12	0.959	0.711	0.589	0.972	0.628	0.525
		167 × 12	0.965	0.602	0.475	0.980	0.476	0.388
	0.5	250 × 12	0.929	0.936	0.765	0.950	0.805	0.674
		200 × 12	0.951	0.682	0.552	0.968	0.522	0.435
		167 × 12	0.951	0.679	0.556	0.967	0.503	0.403
Normal	0.9	250 × 12	0.948	0.893	0.759	0.949	0.873	0.738
		200 × 12	0.978	0.664	0.544	0.980	0.667	0.559
		167 × 12	0.979	0.581	0.509	0.983	0.540	0.468
	0.7	250 × 12	0.977	0.515	0.410	0.979	0.464	0.389
		200 × 12	0.975	0.614	0.524	0.974	0.569	0.500
		167 × 12	0.980	0.568	0.487	0.981	0.483	0.410
	0.5	250 × 12	0.968	0.572	0.454	0.970	0.585	0.483
		200 × 12	0.970	0.539	0.434	0.973	0.541	0.436
		167 × 12	0.970	0.527	0.425	0.975	0.502	0.408

**Table 5:** Results of target model 1.

Membership function	Membership value	Input size	Target model 1						E
			Training			Test			
			R	RMSE	MAE	R	RMSE	MAE	
Cusp	0.9	250 × 12	0.872	1.310	1.125	0.875	1.460	1.142	2.393
		200 × 12	0.869	1.363	1.169	0.887	1.489	1.139	2.406
		167 × 12	0.899	1.523	1.348	0.914	1.300	0.979	2.130
	0.7	250 × 12	0.913	1.303	1.098	0.892	1.376	1.040	2.259
		200 × 12	0.932	1.118	0.953	0.925	1.271	0.944	2.098
		167 × 12	0.912	1.301	1.127	0.904	1.210	0.923	1.979
	0.5	250 × 12	0.952	1.102	0.969	0.933	1.057	0.916	4.966
		200 × 12	0.892	1.301	1.112	0.884	1.364	1.141	5.650
		167 × 12	0.936	1.486	1.343	0.931	1.516	1.241	6.053
Ridge	0.9	250 × 12	0.914	1.373	1.169	0.888	1.495	1.268	2.613
		200 × 12	0.948	1.203	1.044	0.949	1.276	1.098	2.295
		167 × 12	0.948	1.115	0.973	0.943	1.179	1.023	2.138
	0.7	250 × 12	0.952	1.389	1.215	0.958	1.418	1.232	2.700
		200 × 12	0.930	1.315	1.103	0.900	1.418	1.197	2.421
		167 × 12	0.963	0.915	0.792	0.959	0.958	0.826	1.745
	0.5	250 × 12	0.913	1.222	1.014	0.908	1.299	1.070	2.264
		200 × 12	0.942	1.137	0.968	0.928	1.235	1.072	2.212
		167 × 12	0.933	1.288	1.075	0.906	1.336	1.134	2.313
Normal	0.9	250 × 12	0.795	1.470	1.226	0.858	1.444	1.206	2.395
		200 × 12	0.946	0.993	0.808	0.934	1.133	0.938	1.975
		167 × 12	0.960	1.047	0.948	0.949	1.138	1.033	2.095
	0.7	250 × 12	0.834	1.424	1.197	0.871	1.451	1.218	2.377
		200 × 12	0.943	1.170	1.008	0.942	1.250	1.074	2.249
		167 × 12	0.936	1.272	1.104	0.916	1.274	1.121	2.237
	0.5	250 × 12	0.866	1.544	1.323	0.856	1.624	1.394	2.665
		200 × 12	0.962	1.119	0.948	0.949	1.185	0.987	2.117
		167 × 12	0.934	1.079	0.919	0.928	1.160	0.993	2.049

**Table 6:** Results of source model 2.

Membership function	Membership value	Input size	Source model 2					
			Training			Test		
			R	RMSE	MAE	R	RMSE	MAE
Cusp	0.9	250 × 12	0.990	0.513	0.421	0.986	0.557	0.468
		200 × 12	0.992	0.557	0.483	0.991	0.603	0.539
		167 × 12	0.994	0.392	0.335	0.993	0.429	0.390
	0.7	250 × 12	0.986	0.519	0.433	0.982	0.614	0.539
		200 × 12	0.987	0.444	0.358	0.984	0.490	0.416
		167 × 12	0.987	0.537	0.462	0.984	0.650	0.572
	0.5	250 × 12	0.975	0.657	0.549	0.974	0.695	0.608
		200 × 12	0.978	0.495	0.412	0.978	0.498	0.431
		167 × 12	0.978	0.662	0.561	0.981	0.705	0.626
Ridge	0.9	250 × 12	0.983	0.631	0.519	0.985	0.698	0.616
		200 × 12	0.986	0.519	0.430	0.985	0.568	0.485
		167 × 12	0.992	0.452	0.368	0.991	0.531	0.441
	0.7	250 × 12	0.980	0.527	0.419	0.978	0.560	0.491
		200 × 12	0.977	0.515	0.421	0.976	0.540	0.463
		167 × 12	0.984	0.496	0.411	0.984	0.514	0.443
	0.5	250 × 12	0.983	0.562	0.460	0.977	0.614	0.524
		200 × 12	0.983	0.748	0.638	0.974	0.884	0.787
		167 × 12	0.981	0.604	0.418	0.971	0.702	0.600
Normal	0.9	250 × 12	0.983	0.603	0.462	0.984	0.596	0.462
		200 × 12	0.992	0.373	0.270	0.994	0.334	0.255
		167 × 12	0.989	0.447	0.338	0.992	0.407	0.321
	0.7	250 × 12	0.985	0.623	0.529	0.984	0.564	0.498
		200 × 12	0.991	0.474	0.380	0.992	0.425	0.359
		167 × 12	0.992	0.400	0.332	0.992	0.348	0.294
	0.5	250 × 12	0.975	0.712	0.601	0.973	0.795	0.687
		200 × 12	0.985	0.535	0.455	0.984	0.594	0.515
		167 × 12	0.987	0.502	0.405	0.986	0.549	0.470

**Table 7:** Results of target model 2.

Membership function	Membership value	Input size	Target model 2						E
			Training			Test			
			R	RMSE	MAE	R	RMSE	MAE	
Cusp	0.9	250 × 12	0.874	1.406	1.229	0.897	1.414	1.059	2.249
		200 × 12	0.903	1.397	1.223	0.932	1.332	1.024	2.215
		167 × 12	0.819	1.439	1.235	0.810	1.620	1.203	2.302
	0.7	250 × 12	0.903	1.353	1.175	0.885	1.337	0.998	2.104
		200 × 12	0.940	1.172	1.014	0.954	1.162	0.903	2.002
		167 × 12	0.930	1.134	0.982	0.908	1.178	0.871	1.890
	0.5	250 × 12	0.876	1.427	1.234	0.830	1.489	1.216	2.305
		200 × 12	0.960	1.044	0.929	0.962	1.064	0.867	1.900
		167 × 12	0.948	1.178	1.049	0.942	1.193	0.983	2.090
Ridge	0.9	250 × 12	0.938	1.337	1.136	0.942	1.430	1.206	2.522
		200 × 12	0.900	1.272	1.090	0.925	1.332	1.146	2.326
		167 × 12	0.965	1.089	0.968	0.960	1.139	1.009	2.080
	0.7	250 × 12	0.884	1.542	1.290	0.877	1.617	1.350	2.661
		200 × 12	0.969	0.952	0.815	0.969	0.994	0.834	1.813
		167 × 12	0.951	1.321	1.169	0.946	1.406	1.239	2.543
	0.5	250 × 12	0.945	1.045	0.860	0.945	1.108	0.939	1.980
		200 × 12	0.948	1.059	0.873	0.946	1.124	0.956	2.020
		167 × 12	0.962	1.295	1.137	0.958	1.301	1.158	2.427
Normal	0.9	250 × 12	0.940	1.452	1.280	0.944	1.558	1.343	2.782
		200 × 12	0.910	1.319	1.148	0.918	1.424	1.232	2.454
		167 × 12	0.931	1.346	1.184	0.926	1.443	1.250	2.514
	0.7	250 × 12	0.917	1.235	1.070	0.930	1.268	1.077	2.216
		200 × 12	0.955	1.040	0.892	0.956	1.128	0.959	2.012
		167 × 12	0.962	1.052	0.925	0.949	1.167	1.005	2.076
	0.5	250 × 12	0.822	1.529	1.263	0.874	1.508	1.228	2.459
		200 × 12	0.882	1.320	1.105	0.889	1.401	1.178	2.328
		167 × 12	0.953	1.083	0.947	0.953	1.179	1.015	2.120

For the Source model 2–Target model 2, as can be seen from the minimum value 1.813 of *E* in Table 7, transfer learning has the best effect when the membership function is ridge, the membership value is 0.7 and the input size is 200 × 12. For the last transfer learning model (Source model 3–Target model 3), the results are shown in Tables 8 and 9.

**Table 8:** Results of source model 3.

Membership function	Membership value	Input size	Source model 3					
			Training			Test		
			R	RMSE	MAE	R	RMSE	MAE
Cusp	0.9	250 × 12	0.980	0.636	0.495	0.979	0.717	0.553
		200 × 12	0.984	0.514	0.413	0.981	0.575	0.462
		167 × 12	0.982	0.606	0.510	0.982	0.642	0.533
	0.7	250 × 12	0.978	0.476	0.387	0.978	0.511	0.430
		200 × 12	0.980	0.547	0.458	0.979	0.589	0.488
		167 × 12	0.983	0.548	0.460	0.982	0.598	0.513
	0.5	250 × 12	0.959	0.692	0.557	0.958	0.732	0.603
		200 × 12	0.965	0.841	0.742	0.966	0.828	0.723
		167 × 12	0.965	0.594	0.481	0.963	0.612	0.511
Ridge	0.9	250 × 12	0.985	0.513	0.432	0.986	0.536	0.445
		200 × 12	0.983	0.501	0.419	0.983	0.518	0.430
		167 × 12	0.989	0.444	0.381	0.989	0.452	0.388
	0.7	250 × 12	0.972	0.726	0.608	0.965	0.795	0.679
		200 × 12	0.978	0.529	0.416	0.974	0.588	0.462
		167 × 12	0.985	0.534	0.462	0.982	0.602	0.541
	0.5	250 × 12	0.968	0.612	0.495	0.972	0.608	0.492
		200 × 12	0.969	0.601	0.484	0.971	0.630	0.520
		167 × 12	0.973	0.563	0.458	0.975	0.568	0.464
Normal	0.9	250 × 12	0.985	0.447	0.367	0.983	0.487	0.398
		200 × 12	0.980	0.642	0.563	0.979	0.665	0.583
		167 × 12	0.989	0.384	0.300	0.988	0.418	0.319
	0.7	250 × 12	0.981	0.640	0.521	0.979	0.743	0.609
		200 × 12	0.975	0.579	0.474	0.973	0.661	0.528
		167 × 12	0.987	0.489	0.415	0.986	0.521	0.414
	0.5	250 × 12	0.985	0.510	0.416	0.983	0.524	0.426
		200 × 12	0.987	0.478	0.390	0.982	0.500	0.414
		167 × 12	0.988	0.499	0.414	0.986	0.511	0.437

**Table 9:** Results of target model 3.

Membership function	Membership value	Input size	Target model 3						E
			Training			Test			
			R	RMSE	MAE	R	RMSE	MAE	
Cusp	0.9	250 × 12	0.860	1.436	1.231	0.827	1.688	1.276	2.504
		200 × 12	0.835	1.345	1.126	0.826	1.723	1.350	2.587
		167 × 12	0.827	1.490	1.278	0.789	1.609	1.187	2.248
	0.7	250 × 12	0.928	1.298	1.134	0.908	1.344	1.042	2.216
		200 × 12	0.891	1.432	1.203	0.878	1.463	1.170	2.363
		167 × 12	0.884	1.248	1.060	0.863	1.424	1.088	2.208
	0.5	250 × 12	0.906	1.236	1.037	0.885	1.407	1.093	2.309
		200 × 12	0.936	1.170	0.988	0.933	1.321	1.071	2.308
		167 × 12	0.962	1.137	0.963	0.947	1.207	0.958	2.128
Ridge	0.9	250 × 12	0.956	0.994	0.909	0.954	1.118	0.958	2.009
		200 × 12	0.965	1.051	0.891	0.964	1.153	0.985	2.097
		167 × 12	0.962	1.171	1.041	0.957	1.204	1.091	2.221
	0.7	250 × 12	0.841	1.532	1.322	0.876	1.557	1.328	2.619
		200 × 12	0.948	1.182	1.051	0.942	1.231	1.072	2.228
		167 × 12	0.946	1.048	0.883	0.938	1.191	1.000	2.092
	0.5	250 × 12	0.927	1.160	0.945	0.929	1.308	1.089	2.293
		200 × 12	0.904	1.291	1.097	0.909	1.313	1.151	2.307
		167 × 12	0.906	1.271	1.079	0.868	1.400	1.240	2.352
Normal	0.9	250 × 12	0.949	1.366	1.166	0.947	1.435	1.234	2.572
		200 × 12	0.942	1.296	1.084	0.936	1.417	1.193	2.495
		167 × 12	0.956	1.221	1.064	0.957	1.251	1.085	2.262
	0.7	250 × 12	0.930	1.244	1.047	0.933	1.354	1.126	2.364
		200 × 12	0.872	1.377	1.176	0.878	1.493	1.267	2.489
		167 × 12	0.968	1.177	1.023	0.968	1.200	1.054	2.213
	0.5	250 × 12	0.938	1.208	1.021	0.940	1.312	1.104	2.311
		200 × 12	0.949	1.216	1.064	0.936	1.265	1.085	2.238
		167 × 12	0.946	1.147	0.976	0.937	1.218	1.037	2.144

**Figure 12:**
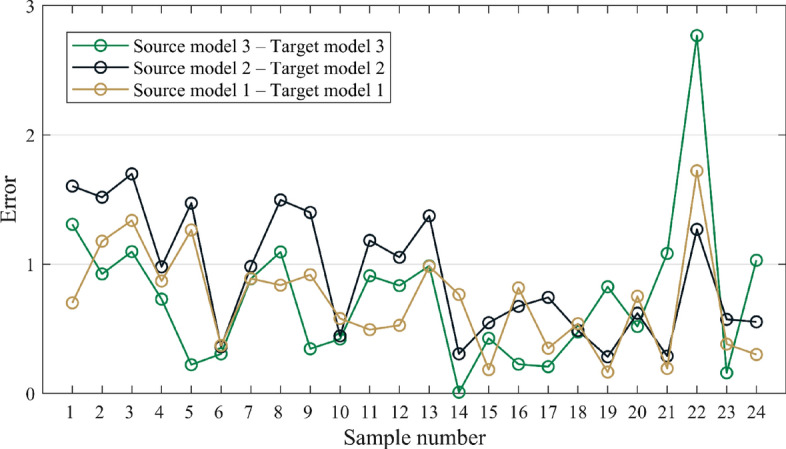
Error contrast.

As can be seen from Table 9, the minimum value of *E* is 2.009, indicating that transfer learning has the best effect when the membership function is ridge, the membership value is 0.9 and the input size is 250 × 12. Among the three transfer learning models, the error indicators (RMSE and MAE) of the target model are larger than that of the source model, and we find that the transfer learning effect will be poor on specific samples, as shown in the Fig. 12.

**Figure 13:**
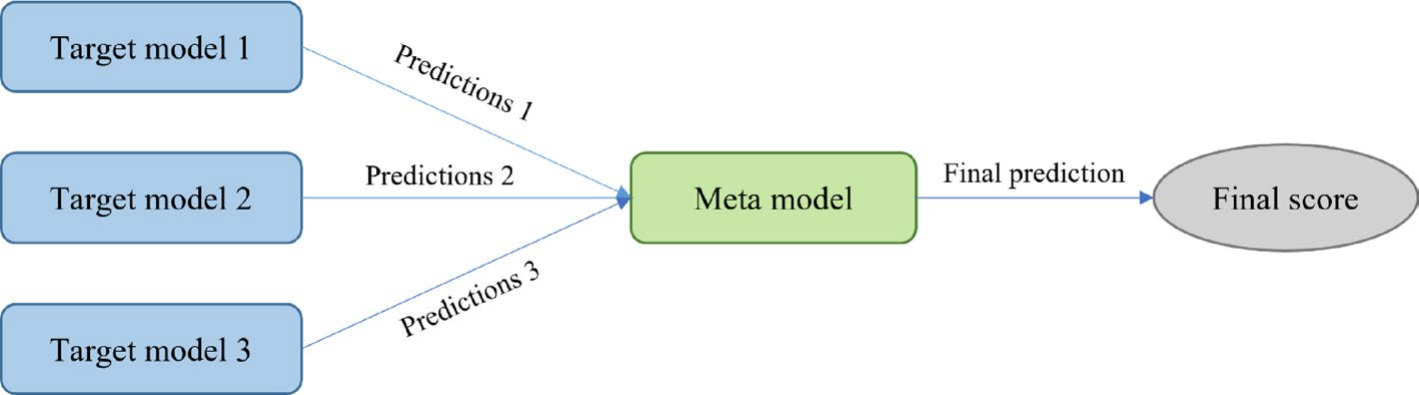
Stacking of three models.

**Figure 14:**
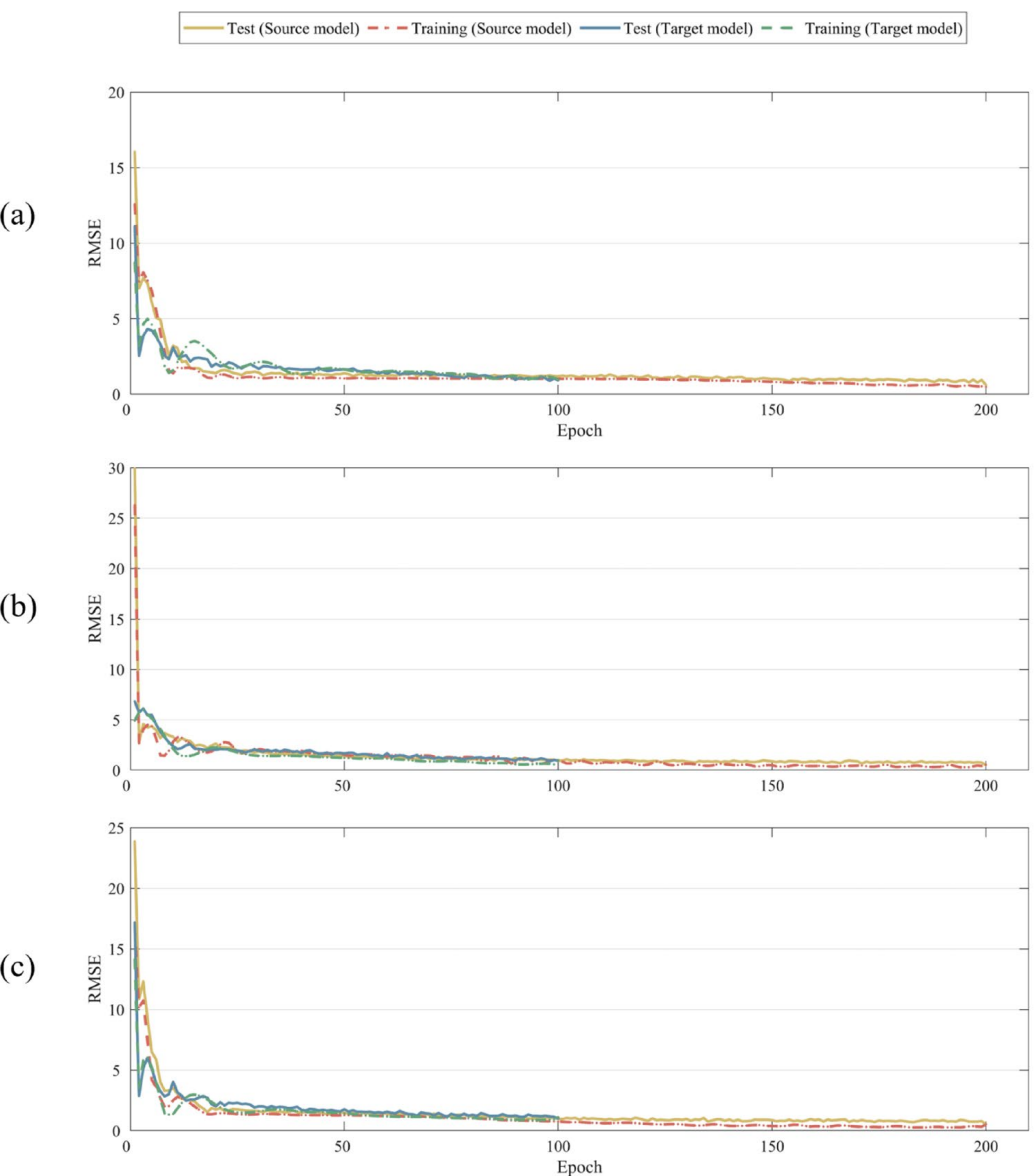
Convergence curve of three models: (**a**) source model 1–target model 1, (**b**) source model 2–target model 2, (**c**) source model 3–target model 3.

In Fig. 12, the performance of the three transfer learning models differs significantly for different samples. To get the most out of each model, the stacking technique is shown in Fig. 13. Firstly, we train the three transfer learning models at their best situation, and Fig. 14 shows their convergence curves. Among the three transfer learning models, the initial RMSE of the target model is significantly smaller than that of the source model. It shows that the iteration of the target model is smoother, unlike the source model which has an obvious period of rapid convergence. The three target models are taken as the base model, and the prediction results of the base model are taken as the training data of the meta model. After training the meta model, the prediction of the base model can be effectively integrated. In this paper, the meta model adopts the method of linear regression, and the regression equation is shown as follows:

17 $$P_{{{\text{final}}}} = - 1.38 + 0.11p_{1} + 0.30p_{2} + 0.97p_{3}$$

where *P*_*final*_ is the final prediction result of the meta model, *p*_*1*_ is the prediction result of Target model 1, *p*_*2*_ is the prediction result of Target model 2, and *p*_*3*_ is the prediction result of Target model 3. Finally, the prediction results of the meta model are shown in the Table 10.

**Table 10:** Final results.

Model	R	RMSE	MAE
Meta model	0.993	0.238	0.181
Target model 1	0.959	0.958	0.826
Target model 2	0.969	0.994	0.834
Target model 3	0.954	1.118	0.958
Lasso	0.977	0.330	0.287
SVR	0.974	0.354	0.270

To compare with the traditional sound quality evaluation methods, we also use lasso regression and support vector regression (SVR)^32,33^. Lasso regression is a linear model, which uses L1 penalty term to control variable selection and complexity. This helps reduce the risk of overfitting and enhances the interpretability of the model in data containing multiple related predictors. There are a large number of potential explanatory variables in sound quality prediction, and lasso regression can help identify which features are most important, simplifying the model and improving prediction performance. Unlike lasso regression, SVR is a nonlinear model that deals with linearly indivisible data by using different kernel functions. There are two main advantages of SVR: it has good robustness to outliers and can work effectively in high-dimensional space. For problems such as sound quality that involves complex nonlinear relationships, SVR can provide powerful modeling capabilities. Based on the six acoustic parameters as inputs and the subjective evaluation scores as outputs, we train the lasso regression model and SVR model. For the lasso regression model, five-fold cross validation is used to find the optimal parameter λ is 68. As for the SVR model with radial basis function, five-fold cross validation is also used to find the optimal parameter, the value range is [0.01, 0.1, 1, 10, 100], and the optimal parameters (penalty parameter *c* = 100 and kernel parameter *g* = 0.01) can be obtained. Due to the small size of the original dataset, we choose the cross-validation approach to get a robust model performance evaluation. Five-fold cross-validation is used as a test method for lasso regression model and SVR model. The prediction results of lasso regression model and SVR model are also shown in the Table 10. Compared with the three target models, the three indicators of the meta model are significantly better, especially the error indexes (RMSE and MAE) are particularly small. Compared with the two traditional methods, the meta model also has obvious advantages: the maximum correlation coefficient R is 0.993, the minimum RMSE is 0.238, and the minimum MAE is only 0.181. Therefore, the results show that the multi-source transfer learning convolutional neural network (MSTL-CNN) proposed in this paper has the best effect and the most accurate results in the evaluation of sound quality.

## Conclusion

In this paper, we do a series of noise tests to establish the sound quality evaluation model of roller chain transmission system. Firstly, 48 noise samples are obtained through noise acquisition test, and all the noise samples are evaluated subjectively and objectively. For the subjective evaluation, the results with poor correlation are excluded. As for the objective evaluation, we calculate the Mel frequency cepstral coefficients and six acoustic parameters.

To solve the problem of small sample sound quality evaluation, we propose a multi-source transfer learning convolutional neural network (MSTL-CNN) based on three source tasks. For the transfer learning model, three membership functions (cusp, ridge and normal) and three membership values (0.9, 0.7 and 0.5) are selected for fuzzy generation. By comparing the transfer learning results in different situations, the optimal conditions of each transfer learning model are found. Since the three transfer learning models behave differently on different samples, we stack their predictions into one meta model. The results of the meta model are not only much better than each transfer learning model, but also better than the traditional methods of sound quality research. In particular, the MSTL-CNN proposed in this paper has the smallest mean absolute error of only 0.181, indicating that the model is the most accurate in the evaluation of sound quality. In the future work, how to remove the stacking steps to simplify the model structure is a research difficulty. Therefore, it is crucial to realize the simultaneous training of three transfer models and timely knowledge sharing for specific samples.

## Data Availability

The datasets generated during and/or analyzed during the current study are available from the corresponding author on reasonable request.
